# Titanium Dioxide Modulation of the Contractibility of Visceral Smooth Muscles In Vivo

**DOI:** 10.1186/s11671-017-1865-7

**Published:** 2017-02-20

**Authors:** Olga V. Tsymbalyuk, Anna M. Naumenko, Oleksandr O. Rohovtsov, Mykola A. Skoryk, Ivan S. Voiteshenko, Valeriy A. Skryshevsky, Tamara L. Davydovska

**Affiliations:** 1 Institute of High Technologies, Taras Shevchenko National University of Kyiv, 2, korp. 4g, Pr. Akademika Hlushkova, Kyiv, 03022 Ukraine; 2 NanoMedTech LLC, 68, Antonovycha Str, Kyiv, 03680 Ukraine

**Keywords:** TiO_2_, Caecum, Myometrium, Contractile activity, High potassium contraction, Acetylcholine, Oxytocin, Diazoxide, Glibenclamide, Elemental analysis

## Abstract

Electronic scanning microscopy was used in the work to obtain the image and to identify the sizes of titanium dioxide (TiO_2_) nanoparticles 21 ± 5 nm. The qualitative and quantitative elemental analysis of the preparations of the *caecum*, antrum, myometrium, kidneys, and lungs of the rats, burdened with titanium dioxide, was also performed. It was established using the tenzometric method in the isometric mode that the accumulation of titanium dioxide in smooth muscles of the caecum resulted in the considerable, compared to the control, increase in the frequency of their spontaneous contractions, the decrease in the duration of the contraction–relaxation cycle, and the decrease in the indices of muscle functioning efficiency (the index of contractions in Montevideo units (MU) and the index of contractions in Alexandria units (AU)). In the same experimental conditions, there was not the increase, but the decrease in the frequency of spontaneous contractions, the duration of the contraction–relaxation cycle, and the increase in MU and AU indices in the smooth muscles of myometrium (in the group of rats, burdened with TiO_2_ for 30 days). It was also determined that TiO_2_ modulates both the mechanisms of the input of extracellular Ca^2+^ ions and the mechanisms of decreasing the concentration of these cations in smooth muscle cells of the caecum during the generation of the high potassium contraction. In these conditions, there is a considerable increase in the normalized maximal velocity of the contraction phase and the relaxation phase. It was demonstrated in the work that titanium dioxide also changes the cholinergic excitation in these muscles. The impact of titanium dioxide in the group of rats, burdened with TiO_2_, was accompanied with a considerable impairment of the kinetics of forming the tonic component of the oxytocin-induced contraction of the smooth muscles of myometrium.

## Background

At present, the elaboration of materials with principally new characteristics is related to obtaining nanosize systems, in particular, nanosize material—the amphoteric compound of TiO_2_, the world market of which has been recently estimated as approximately five million tons annually, a large sector being taken by food, medical, and pharmacological industries [1–3]. Still, there is an urgent problem of TiO_2_ toxicity for living organisms, which is getting even more complicated by its high activity in the interaction with other chemical substances and, due to photocatalytic properties [4–6], with physical factors. Common modifications of TiO_2_ are tetragonal structures: rutile and anatase. Titanium dioxide is remarkable for adsorption, acid-alkaline properties (superficial properties of nanoparticles) which are considerably dependent on pH of the medium. Most acid centers with pК = 3.46 and 4.1 of the surface of TiO_2_ nanoparticles are formed in the acid medium, whereas most alkaline ones with pК = 7.4—in the neutral medium, which is related to the ability of titanium to form different pH-dependent amphoteric forms of hydroxides: TiO_2_·2H_2_O; TiO(OH)_2_; Ti(OH)_4_, etc. [7], which should be taken into consideration in the experimental studies in vitro. As stated above, titanium dioxide is a polar adsorbent. Its active centers are superficial hydroxyl groups, capable of dissociating and forming positively and (or) negatively charged adsorption centers [8]. Recent investigations have demonstrated [9–12] that such active centers of TiO_2_ are capable of interacting with thiol, carboxyl groups, and aminoacids and groups of side chains of aminoacids (in a lesser degree), as well as with peptide bonds of protein macromolecules, in particular, membrane receptors of excited cells. At present, our studies [13] and investigations of other authors [14] using the method of molecular docking of nanosize titanium dioxide material to the extracellular domain of a number of receptors have determined potentially probable sites of binding for this nanosize material of different affinity and analyzed the character of bonds, stabilizing them according to their aminoacid composition. It is known from the scientific literature [15, 16] that receptor-governed intracellular signaling cascades, for instance, those of smooth muscles, play an important role in the regulation of the functions of gastrointestinal tract, myometrium, etc. It was demonstrated in our works [17, 18], conducted in vitro using the isolated preparations of the stomach, circular smooth muscles of the caecum, and myometrium of rats, that the targets of titanium dioxide suspension with the size of nanoparticles of 21 ± 5 nm may be receptor-dependent regulatory mechanisms of smooth muscle cells. Due to this fact, it was interesting to study the state of the mentioned regulatory mechanisms in conditions of the burden of titanium dioxide suspension on rats and to conduct the elemental analysis of these preparations by the quantitative method, using atomic emission spectrometer ICP AES Shimadzu–9000, which has become the aim of our studies.

## Methods

The 8-week-old Wistar rats of both genders were used for the experiments in vivo. The rats were kept in standard conditions of the vivarium (room temperature of 20 ± 2 °C, relative humidity 50–70%, light–dark cycle 12:12 h). TiO_2_ nanoparticles were dispersed in the distilled water using ultrasound for 15 min; to obtain the homogeneous suspension, the latter was additionally stirred with the mechanical stirrer prior to each use. The intragastral dose was selected according to the data about the intake of TiO_2_ with food products in Great Britain: 5 mg per person daily, which is the equivalent of 0.1 mg/kg of weight a day [19]. Due to this, every day, the rats were intragastrically introduced to the suspension of TiO_2_ with the consideration of 0.1 mg/kg in the first experimental group for 30 days and in the second experimental group for 100 days. The bodyweight of rats was estimated every 4–6 days.

All manipulations with animals were carried out in accordance with The International Convention of working with animals and Ukraine Law “On animals protection from cruel treatment” and the minutes of the meeting № 2 of the Bioethical Committee of ERC “Institute of Biology and Medicine” of Taras Shevchenko National University Kyiv from October 20, 2016. Killing of animals was performed via the introduction of lethal dose of anesthetic propofol (Sigma).

The experiments were conducted using the isolated preparations of circular smooth muscles of the caecum and myometrium of rats, burdened with the suspension of TiO_2_. The abduction of spontaneous contractibility of smooth muscle stripes (SMS) of the caecum and myometrium was conducted by the tenzometric method in the isometric mode with the following calculations: the frequency of contractions for 10 min; the average value of contraction–relaxation cycle; the duration of specific fragments of contractions (contraction phase, relaxation phase); the asymmetry coefficient; the index of contractions in Montevideo units; and the index of contractions in Alexandria units. The method [20, 21] was also used to conduct the kinetic analysis of spontaneous contractions-relaxations of muscle preparations and the ones induced by high potassium Krebs solution, acetyl choline, and oxytocin (also in the presence of blocking agents) with the estimation of normalized maximal velocities of contractions (V_nc_)–relaxations (V_nr_).

The normal Krebs solution (NKS) was used in the experiments with the following concentration of components (in mmol/l): NaCl, 120.4; KCl, 5.9; NaHCO_3_, 15.5; NaH_2_PO_4_, 1.2; MgCl_2_, 1.2; CaCl_2_, 2.5; glucose, 11.5; and pH 7.4. The high potassium Krebs solution with the concentration of К^+^ ions (80 mmol/l) was prepared by replacing the required amount of Na^+^ ions in the standard Krebs with the equimolar amount of К^+^ ions. The substances were used in the following concentrations: acetyl choline (AC), 10^−5^ mol/l (Soyuzkhimreaktyv, Russian Federation); oxytocin, 0.1 IU (Gedeon-Richter, Hungary); activator of ATP-sensitive К^+^-channels, diazoxide (Sigma); blocking agent for ATP-sensitive К^+^-channels of the plasmatic membrane, glibenclamide (Sigma), 10^−5^ mol/l; blocking agent for ATP-sensitive К^+^-channels of mitochondria—5-hydroxydecanoate (5-HD, Sigma), 2·10^−4^ mol/l.

The statistical analysis of the experimental results was performed using the program OriginPro 8. The unpaired version of Student’s *t* test was used to determine the reliable differences between the mean values of two samplings. Multiple comparisons were performed using the parametric one-factor dispersion analysis. The results were considered reliable on the condition of the probability value of *p* under 5% (*p* < 0.05). The results were presented as the arithmetic mean ± standard error of the mean value, *n*—number of experiments.

**Fig. 1:**
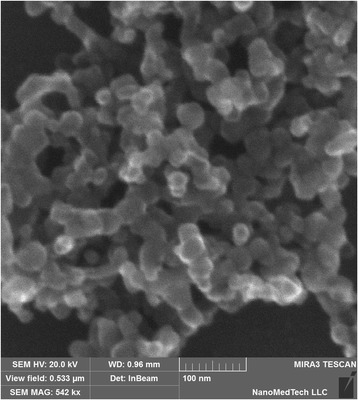
Micro-picture of titanium dioxide nanoparticles

The nanoparticles of TiO_2_ (PlasmaChem GmbH, D-12489 Berlin, Germany) were used in the form of nanopowder (mixture of rutile and anatase) with the average size of particles of 21 ± 5 nm (the measurements were conducted using the scanning electronic microscope *Tescan Mira 3 LMU*, kindly provided for our work by NanoMedTech LLC) (Fig. 1); specific area, 50 ± 10 m^2^/g; purity >99.5%; content of Al_2_O_3_ <0.3 wt; SiO_2_ <0.2 wt. The nanopowder of TiO_2_ was previously resuspended in dimethylsulfoxide (DMSO) assuming the presence of 0.25% DMSO in the final volume. Likewise all the control solutions contained 0.25 DMSO. The destruction of the aggregates of TiO_2_ nanoparticles in the suspension was performed using the ultrasound processing for 2 min at the frequency of 37 kHz. The zeta-potential of TiO_2_ nanoparticles suspension, estimated using the Zetasizer nano device (kindly provided by NanoMedTech LLC), was −7.93 mV.

The qualitative and quantitative elemental analysis of smooth muscle preparations of the caecum, uterus, antrum, kidneys, lungs, and liver of rats, burdened with the suspension of nanosize titanium dioxide material (10^−2^ mg/ml), was performed in the work using the atomic emission spectrometer ICP AES Shimadzu –9000, kindly provided by NanoMedTech LLC. The weighed quantities were placed in polytetrafluoroethylene (PTFE)—a high-pressure hose. The preparations were added 10 ml of HNO_3_ of 65% ACS qualification. The samples were heated in the microwave device for 20 min and kept at the temperature of 200 °C for 20 more min. After cooling, the mineralized samples were transferred to the measuring vials of 100 cc; the volume of the solution was enlarged to the mark using class I water (18 MOm/cm) with subsequent analysis of solutions using the abovementioned atomic emission spectrometer. The conditions of spectrum registration were as follows: ignition mode, normal (water); attached instruments, mini torch; radio frequency power, 1.20 kW; plasma gas, 10.00 l/min; auxiliary gas, 0.60 l/min; carrier gas, 0.70 l/min; exposure time, 30 s; condition, wide range; and instrument name, B41844700444.

## Results and Discussion

**Fig. 2:**
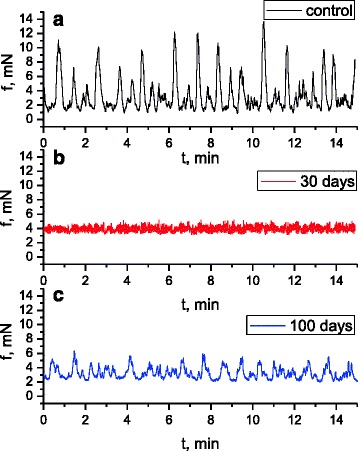
Spontaneous contractive activity of circular smooth muscles of the caecum of the rats in the control group (**a**) and the group of rats, burdened with the suspension of titanium dioxide for 30 days (**b**) and 100 days (**c**)

**Fig. 3:**
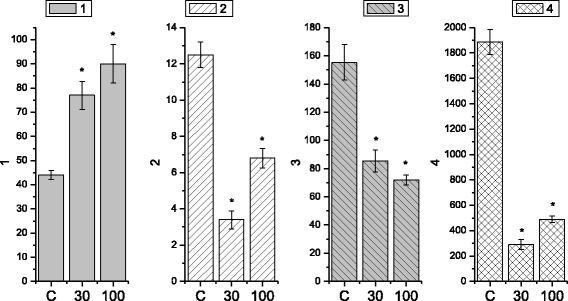
Histograms of the kinetic analysis parameters for spontaneous contractive activity of circular smooth muscles of the caecum (*C*, control; 30 and 100, term (days) of burdening the rats with the suspension of nanosize titanium dioxide material): (1) frequency of preparation contractions for 10 min; (2) averaged value of the duration of contraction–relaxation cycle (sec); (3) MU index of contractions; and (4) AU index of contractions. *p < 0.05, reliability of changes compared to the control

It is known [22, 23] that the electric and contractive activity of visceral smooth muscles occurs via parasympathetic and sympathetic control with the direct participation of excitation and inhibition neuromediators, respectively. At the same time, even with no impact of the neuromediators, hormones in smooth muscles, for instance, in stomach, there is registered electric and relevant contractive activity, the formation of which is based on the mechanisms of pacemaking activity of interstitial cells of Cajal [24, 25]. In our work, the tenzometric method in the isometric mode was used to investigate the spontaneous contractive activity of isolated smooth muscle stripes of the caecum of three groups of rats: control group (*n* = 6) and two groups of animals (6 per each), which were burdened with the suspension of nanosize titanium dioxide material in the abovementioned concentration for 30 and 100 days respectively. The duration of the registration of spontaneous contractions in the two groups was 60 min. During the mentioned time period, the level of SMS basal tone remained stable. The measurements, conducted in the control group (Fig. 2a), demonstrated that the amplitude of spontaneous contractions of the muscle preparations changed in the range from 1 to 12.5 mN, *n* = 12. The indicated interval of amplitudes demonstrated their distribution by frequencies with two reliable maximums: 10.9 ± 0.73%, *n* = 12 with the amplitude of 3 mN and the shift along the scale of amplitudes towards their increase, 5.45 ± 0.37%, *n* = 12, (amplitude of 8.5 mN). Here, the contraction of muscle preparations with low amplitude was observed more frequently, compared to the contractions with high amplitude, which is in good agreement with the literature data [26, 27]. The frequency of preparation contractions, estimated in the control (Fig. 3) for 10 min, was equal to 44 ± 1.8; the average value of contraction–relaxation cycle was 12.5 ± 0.7 s; the duration of specific fragments of contractions was as follows: contraction phase, 5.2 ± 0.4 s; relaxation phase, 7 ± 0.4 s; asymmetry coefficient, 1.19 ± 0.07; index of contractions in Montevideo units (MU), 155.3 ± 12.6; and index of contractions in Alexandria units (AU), 1887.138 ± 96.9.

The studies, conducted with smooth muscle stripes of the caecum, burdened with the suspension of nanosize material of TiO_2_ for 30 days, demonstrated a considerable decrease, compared to the control, in the amplitude of spontaneous contractions (the range from 0.5 to 2 mN) (Fig. 2b), and the narrowing of their amplitude–frequency range, within which the maximum values, were shown by the contractions with the frequency of 10.3 ± 0.83%, *n* = 12. The frequency of preparation contractions, estimated in the control (Fig. 3) for 10 min, equaled 77 ± 5.8, which exceeded the control value more than 1.5-fold; the averaged value of the contraction–relaxation cycle was 3.4 ± 0.5 s, which was three times less than the control; the duration of specific contraction fragments was as follows: contraction phase,1.49 ± 0.1 s, and relaxation phase, 1.91 ± 0.04 s, which was three times less than the control; asymmetry coefficient, 0.87 ± 0.06, which was twice less than the control; MU index of contractions, 85.4 ± 7.9, which was almost twice less than the control; and AU index of contractions, 290.3 ± 37.8, which was six times less than the control value of this parameter.

It was determined that the main frequency maximums for spontaneous contractions of SMS of rats (Fig. 2c), burdened with the suspension of TiO_2_ in the abovementioned concentration for 100 days, were in the range of 1.25–2.8 mN and had the following values: 12.2 ± 0.9%, *n* = 12 with the amplitude of 1.2 mN and 9.8 ± 0.7%, *n* = 12 with the amplitude of 2 mN. The frequency of contractions, estimated in these experimental conditions for 10 min (Fig. 3), equaled 90 ± 7.9, which exceeded the control value of this parameter more than twofold; the averaged value of the contraction–relaxation cycle was 6.8 ± 0.54 s, whereas in the control, this value exceeded 12 s.; the duration of specific contraction fragments was as follows: contraction phase, 3.2 ± 0.2 s, which was 1.5 times less than the control; relaxation phase, 3.6 ± 0.12 s, which was almost twice less than the control value; asymmetry coefficient, 1.4 ± 0.1; MU index of contractions, 72 ± 3.5, which exceeded the control twice; and AU index of contractions, 489.4 ± 26, which was almost four times less than the control.

Therefore, the differences in the amplitude–frequency characteristics of spontaneous contractive activity of smooth muscles of the caecum and the differences in the parameters of temporal redistribution of its contraction–relaxation phases, asymmetry coefficients, MU and AU indexes, etc., observed in the experimental groups of rats compared to the control, may be related to the ability of nanosize titanium dioxide material to modulate the pacemaking activity of the interstitial cells; the mechanism of which, as stated above according to the scientific literature [25, 28], is determined by the release of calcium ions from the sarcoplasmic reticulum and their adsorption by mitochondria; the fluctuation frequency of their concentration is of the same order, observed for pacemaker currencies; the amplitude–frequency characteristics of which define the parameter values of spontaneous contractions of smooth muscles.

**Fig. 4:**
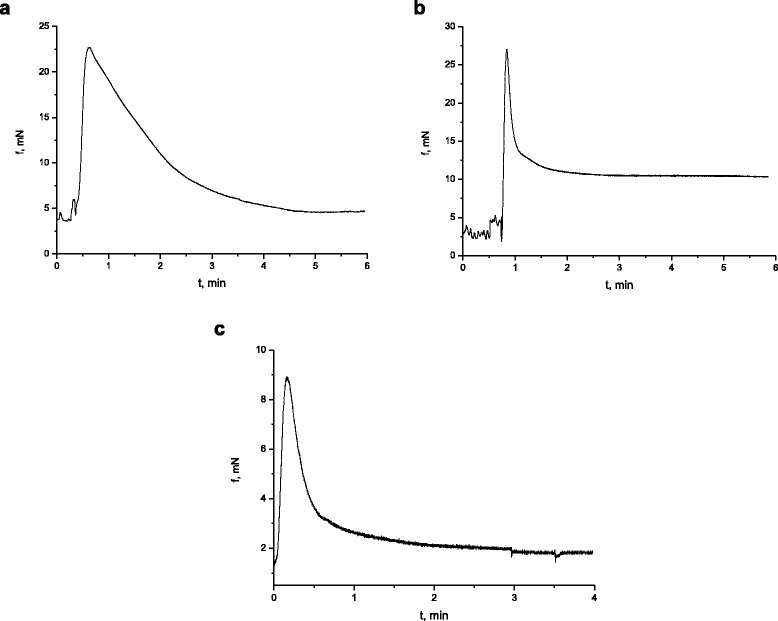
High potassium (80 mmol/l) contraction of circular smooth muscles of the caecum of the rats in the control group (**a**) and the group of rats, burdened with the suspension of titanium dioxide for 30 days (**b**) and 100 days (**c**)

The following series of experiments was aimed at the study of the change in the high potassium contraction of circular smooth muscles of the caecum of rats, burdened with nanosize titanium dioxide material in the abovementioned concentration. In response to the application of high potassium (80 mmol/l) Krebs solution, the muscle preparations of the control group of rats developed contractions–relaxations (Fig. 4a), and the averaged value of phase component of which was 18.8 ± 1.6 mN, *n* = 12, and the ratio of the phase component to the tonic one, (1.03 ± 0.1). The kinetic analysis of К^+^-induced contractions of muscle preparations of the control group of rats demonstrated that in these conditions the normalized maximal velocity of the contraction phase (V_nc_) was 6.7 ± 0.8/min, whereas the normalized velocity of the relaxation phase (V_nr_), 1.38 ± 0.2/min. Figure 4b presents the high potassium contraction of the muscle preparation of rats, burdened with titanium dioxide for 30 days. It was determined that the impact of this nanosize material led to the observed increase in the value of the phase component of contraction compared to the control group of rats by 31.8 ± 1.8%, *n* = 12, and the increase in the normalized maximal velocity of the contraction phase of SMS, the value of which was 17 ± 1.5/min, compared to the control group of rats. The normalized maximal velocity of the relaxation phase was 4.2 ± 0.5/min. Here, the average value of the relative effect for V_nc_ was 253.3% and for V_nr,_ 304.3%. Both parameters exceeded the control values more than twice. The experiments demonstrated the increase in the ratio of the phase component to the tonic one. This ratio was (1.6 ± 0.2) contrary to the control values, which, as stated above, were 1.07 ± 0.1. It is known from the scientific literature [28] that the phase component of the high potassium contraction of smooth gastric muscles, induced by the high potassium solution, is conditioned, on the one hand, by the input of extracellular Ca^2+^ ions via potential-regulated calcium channels and on the other hand, by the activation of the mechanism of induced release of Ca^2+^, which is independent from these cations, from inositol triphosphate- and rianodine-sensitive depot of the sarcoplasmic reticulum of smooth muscle cells of the intestines, the modulation of which by nanosize titanium dioxide material may be the reason of the changes in the parameters of the phase component of the high potassium contraction, registered by us in the experimental group of rats. As for the considerable decrease in the tonic component of the high potassium contraction of this group of rats, registered in the presence of titanium dioxide, it may be assumed based on the works of other authors [29] that it may be caused by titanium dioxide modulating the mechanisms, ensuring both the decrease in the intracellular concentration of calcium ions, and the mechanisms of releasing neuromediators from the nervous terminals of the intramural nervous system of intestines.

Another study was aimed at investigating the changes in the parameters of potassium (80 mmol/l)-induced contraction–relaxation of circular smooth muscles of the caecum of the group of rats (*n* = 6), burdened with the suspension of titanium dioxide nanosize material in the abovementioned concentration for 100 days. It was determined (Fig. 3c) that, contrary to the group of rats, burdened with TiO_2_ for 30 days, there was a decrease in the value of the phase component of the high potassium contraction of muscle preparations by 42.3 ± 3.6%, *n* = 12, *p* < 0.05, compared to the control. In these experimental conditions, the ratio of the phase component and the tonic one was practically identical to the control. The kinetic analysis of the curves of high potassium contraction with the estimates of normalized maximal velocities of separate contraction and relaxation phases demonstrated that such parameter as V_nc_ acquired the value of 8.5 ± 0.74/min, which corresponded to the control level, whereas such parameter as V_nr_ exceeded the control almost twice.

**Fig. 5:**
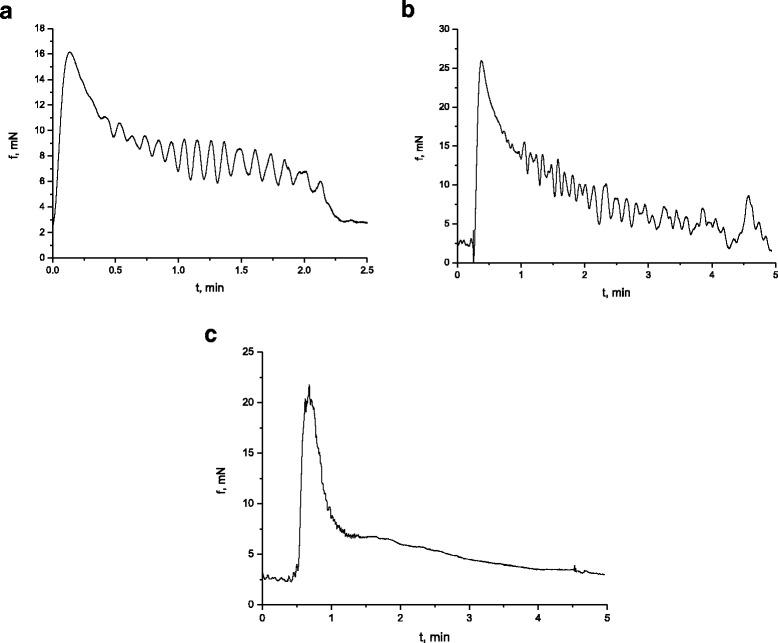
Acetylcholine-induced (10^−5^ mol/l) contraction of circular smooth muscles of the caecum of the rats in the control group (**a**) and the group of rats, burdened with the suspension of titanium dioxide for 30 days (**b**) and 100 days (**c**)

It is known [15, 16] that the parasympathetic control of the contractive activity of the visceral smooth muscles occurs with the involvement of acetylcholine, the excitation neuromediator. Taking this fact into consideration, the contraction of preparations of circular smooth muscles of the caecum, activated by acetylcholine, the agonist of muscarinic receptors, in the concentration of 10^−5^ mol/l, was registered in the work. It was determined (Fig. 5a) that the averaged value of the phase component of the acetyl choline contraction of SMS in the control group of rats (*n* = 6) was 15 ± 0.9 mN, *n* = 12, and its ratio to the tonic component, 1.5 ± 0.1. Here, the estimated normalized maximal velocity of the contraction phase was 8.5 ± 0.6/min, whereas the normalized maximal velocity of the relaxation phase was 1.04 ± 0.4/min. There was also the study of acetylcholine-induced contractions of circular smooth muscles of the caecum (in the abovementioned concentration) of the group of rats (*n* = 6), which were burdened with the suspension of nanosize titanium dioxide material for 30 days (Fig. 5b). It was determined that compared to the control group of animals, there was an increase in the phase component of the acetylcholine contraction of SMS by 70 ± 5%, *n* = 12, *p* < 0.05. At the same time, the averaged value of the ratio of the phase contraction of smooth muscle stripes to the tonic one decreased, compared to the control, and was 1.1 ± 0.08, *n* = 12, *p* < 0.05. The normalized maximal velocity of the contraction phase and the relaxation phase did not differ from the control. It is known [15, 30] that the initiation and maintenance of acetylcholine-induced contraction of circular smooth muscles of the intestines require the increase in the intracellular concentration of Ca^2+^ ions due to the release of these cations from the intracellular depots of their storing and the phosphorylation of myosin light chains (MLC), the level of increasing the activity of MLC or inhibiting the activity of the myosin phosphatase [31]. In our experiments, the phase component of the contraction of muscle preparations of TiO_2_-burdened rats, activated with acetylcholine, increased, which may be caused by the modulating impact of this nanosize material on the abovementioned regulatory mechanisms of cholinergic excitation. At the same time, these experiments demonstrated the approximation of the tonic component of the acetylcholine contraction to the value of its phase component. The reason for such a decrease in the tonic component, when the mechanism of its formation is considered [15, 16], may be the impact of titanium dioxide on the function of muscarinic M2 type receptors, which is based on the restriction of the input of extracellular calcium ions into SMS via potential-regulated calcium channels of L-type.

The study of the cholinergic excitation, caused by acetylcholine in the concentration of 10^−5^ mol/l, was conducted with the group of rats (*n* = 6), burdened with titanium dioxide in the abovementioned concentration for 100 days, and it demonstrated that in these conditions, there was no statistically reliable changes in the value of the phase component of the contraction of smooth muscle stripes of the caecum, whereas its tonic component increased practically to the level of the phase component, similar to the group of TiO_2_-burdened rats for 30 days (Fig. 4c). There were no changes in the normalized maximal velocity of the contraction phase, whereas the velocity of the relaxation phase increased three times compared to the control.

**Fig. 6:**
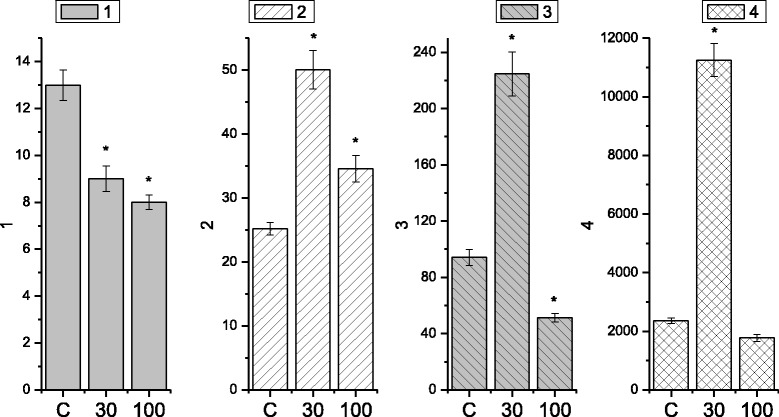
Histograms of the kinetic analysis parameters for spontaneous contractive activity of circular smooth muscles of the caecum (*C*, control; 30 and 100, term (days) of burdening the rats with the suspension of nanosize titanium dioxide material): (1) frequency of preparation contractions for 10 min; (2) averaged value of the duration of contraction–relaxation cycle (sec); (3) MU index of contractions; and (4) AU index of contractions. *p < 0.05, reliability of changes compared to the control

The following series of experiments was dedicated to the study of the impact of nanosize titanium dioxide material on the spontaneous contractive activity of smooth muscles of myometrium (Fig. 6) of rats according to the scheme, similar to the one, described above (experiments using SMS of the caecum). The duration of the registration of spontaneous contractions in the two groups was 60 min. During the mentioned time period, the level of SMS basal tone remained stable. The measurements, conducted in the control group of rats, demonstrated that the amplitude of spontaneous contractions of the isolated smooth muscle stripes was in the range from 1 to 6.5 mN, *n* = 12 with two reliable maximums: 11.1 ± 0.67% with the amplitude of 4.7 mN and 16.7 ± 0.9% with the amplitude of 4.9 mN. The estimation of mechanic and kinetic parameters of such contractions demonstrated that the frequency of contractions of muscle preparations for 10 min was 13 ± 0.7; the averaged value of the contraction–relaxation cycle was 25.2 ± 1.01 s; the duration of pauses between separate contractions was 22 ± 1.4 s; the duration of specific contraction fragments was as follows: contraction phase, 9.07 ± 0.36 s; relaxation phase, 15.8 ± 0.95 s; asymmetry coefficient, 1.15 ± 0.05; MU index of contractions, 94.1 ± 6; and AU index of contractions, 2364.3 ± 94.5.

The estimation of similar mechanic and kinetic parameters of spontaneous contractive activity of myometrium muscle preparations of rats, burdened with TiO_2_ suspension for 30 days, demonstrated that in the range of amplitudes, which was considerably extended compared to the control, from 3 to 18 mN (*n* = 12) with the reliable maximum 17.6 ± 1% (amplitude 14.5 mN), the frequency of contractions of muscle preparations for 10 min was 9 ± 0.5; the averaged value of the duration of contraction–relaxation cycle was 50.1 ± 3 s; the duration of pauses between separate contractions 22.9 ± 1.6 s; the duration of separate contraction fragments was as follows: contraction phase, 18 ± 9 s; relaxation phase, 32.1 ± 1.3 s; asymmetry coefficient, 0.65 ± 0.04; MU index of contractions, 224.6 ± 15.7; and AU index of contractions, 11254.9 ± 562.7.

It was determined that the group of rats with longer (100 days) period of being burdened with the suspension of nanosize titanium dioxide material in the abovementioned concentration, compared to the control, did not demonstrate any shift in the range of the amplitude of spontaneous contractions 1.5–5.8 mN, *n* = 12, and the reliable maximums were as follows: 7 ± 0.4% with the amplitude of 1.7 mN and 6.9 ± 0.4% with the amplitude of 3.5 mN. The estimation of the mechanic and kinetic parameters of spontaneous contractive activity of muscle preparations of the myometrium of rats, burdened with the suspension of TiO_2_ for 100 days, demonstrated that the frequency of contractions of the muscle preparations for 10 min was 8 ± 0.32; the averaged value of the contraction–relaxation cycle was 34.6 ± 2 s; the duration of pauses between separate contractions was 2 ± 0.12 s; the duration of specific contraction fragments was as follows: contraction phase, 10.5 ± 0.6 s; relaxation phase, 24 ± 1.6 s; asymmetry coefficient, 0.7 ± 0.03; MU index of contractions, 51.3 ± 3; AU index of contractions, (1775 ± 124.8).

**Fig. 7:**
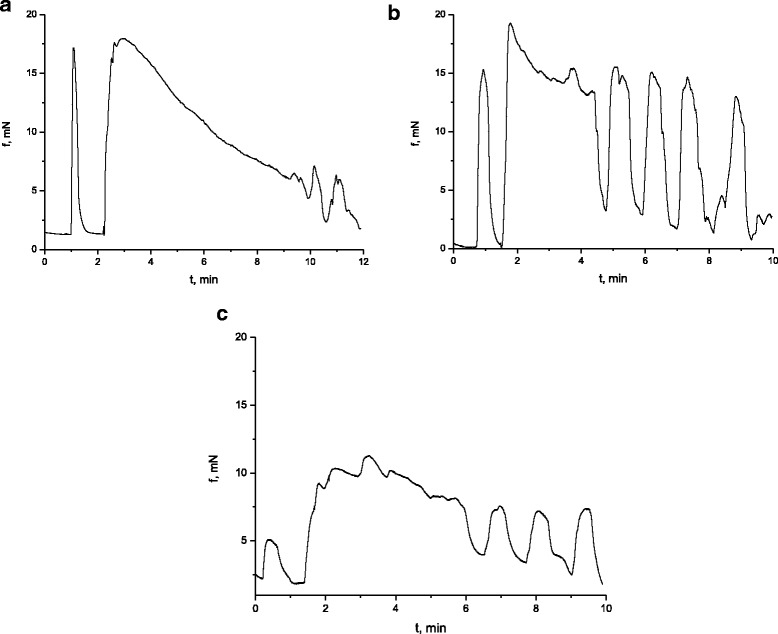
Oxytocin-induced (0.1 IU) contraction of smooth muscles of the myometrium of the rats in the control group (**a**) and the group of rats, burdened with the suspension of titanium dioxide for 30 days (**b**) and 100 days (**c**)

Similar to muscarinic cholinergic receptor-dependent activation of the intracellular signaling cascades in smooth muscle cells of the gastrointestinal tract, the receptor-dependent activation of intracellular signaling cascades of myometrium cells with the participation of uterotonic peptide hormone, oxytocin, is associated with Gq/11-proteins, and their stimulation leads to the increase in the synthesis of secondary mediators, inositol-1,4,5-triphosphate and diacylglycerol, and as a result to the changes in the intracellular concentration of Ca^2+^ ions [32–35]. Taking the abovementioned into consideration, the oxytocin-induced contractions of myometrium SMS of the rats in the control and experimental groups were studied. In the control group of rats (*n* = 6), the introduction of oxytocin (0.1 IU) to standard Krebs solution caused the contraction of SMS of the myometrium (Fig. 7a), the averaged value of the phase component of which was 16.7 ± 1 mN, *n* = 12; the ratio of the value of the phase contraction component to the tonic one was 1.3 ± 0.8. The analysis of the kinetic properties of contractions with the estimation of normalized velocities of separate phases of contraction and relaxation demonstrated that V_nc_ = 6.4 ± 0.5/min, while V_nr_ = 1.3 ± 0.3/min^−1^.

The study of the abovementioned parameters of the isolated SMS of the myometrium of rats in the experimental group (the period of burdening the rats with titanium dioxide was 30 days) demonstrated (Fig. 7b) that, compared to the control, their phase component remained unchanged, 18.3 ± 1.2 mN, *n* = 12, and *p* < 0.05, whereas the tonic component decreased considerably (the ratio of the phase component of such contractions to the tonic one was 4.9 ± 0.3, *n* = 12, *p* < 0.05). The normalized velocity of the contraction and relaxation phases increased almost twice with the values of 11 ± 0.7 min^−1^ and 5.2 ± 0.6 min, respectively. The mentioned changes in the parameters were statistically reliable: the average values of the relative effect were 174.3% and 392.9%, respectively.

**Fig. 8:**
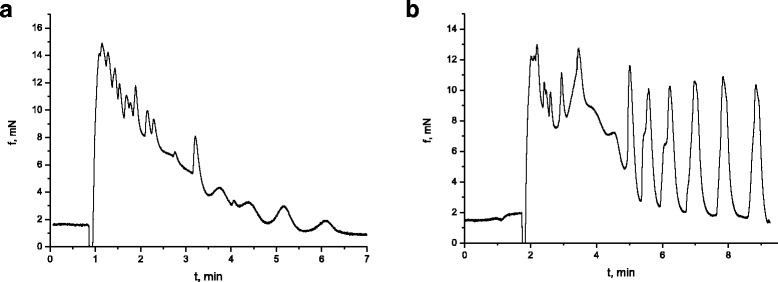
Oxytocin-induced (0.1 IU) contractions of the myometrium SMS in the presence of glibenclamide (10^−5^ mol/l) and 5-hydroxydecanoate (2·10^−4^ mol/l) (**a**) and the same in the presence of titanium dioxide (10^−5^ mg/ml) (**b**)

In condition of longer impact of titanium dioxide (the period of burdening the rats lasted 100 days) on the smooth muscles of myometrium (Fig. 7c), there was a double decrease in the amplitude of the phase contraction component, induced by oxytocin, compared to the control. There was also a double decrease in the normalized velocity of such contractions and more than fivefold decreases in the normalized velocity of its relaxation. As for the tonic component, its formation was delayed with gradual and long-term approximation to the basal level. One of the reasons of the above described changes in the oxytocin-induced contractions of the myometrium muscle preparations of rats, burdened with titanium dioxide, may be the impairment of ATP-dependent mechanisms of the regulation of the conductivity of both plasmatic membrane and the membrane of mitochondria of smooth muscle cells. The experiments in vitro demonstrated oxytocin-induced (0.1 IU) contractions of the myometrium muscle contractions in the control at the impact of glibenclamide, the blocking agent of ATP-sensitive К^+^-channels of the plasmatic membrane of SMS, in the concentration of 10^-5^ mol/l, and the blocking agent of ATP-sensitive К^+^-channels of mitochondria—5-HD in the concentration of 2°10^-4^ mol/l. It was determined that in the presence of the blocking agents in standard Krebs solution, there was an inconsiderable increase in the phase component of oxytocin-induced SMS contraction, compared to the control. The ratio of the phase component to the tonic one remained in the range of the control. At the background of the impact of blocking agents, the titanium dioxide nanosize material in the concentration of 10^−5^ mg/ml was introduced to the solution. On the 20^th^ min of its application, oxytocin was introduced to the suspension. As seen in Fig. 8, the presence of the blocking agents of ATP-sensitive potassium channels of the plasmatic membrane and the membrane of mitochondria in the solution did not remove the inhibiting impact of TiO_2_ on the mechanisms of forming the tonic component of the oxytocin-induced contraction. Taking the abovementioned into consideration along with the results of our previous studies [17, 18], it is likely that there is some impact of titanium dioxide on the smooth muscles of the myometrium along with the membrane and intracellular mechanisms of regulating the intracellular concentration of calcium ions as well as processes, related to the synthesis of adenosine triphosphoric acid in mitochondria. There is also probable involvement of the intramural nervous system in the abovementioned effects of the impact of the nanosize material of TiO_2_ on the smooth muscles of myometrium.

**Fig. 9:**
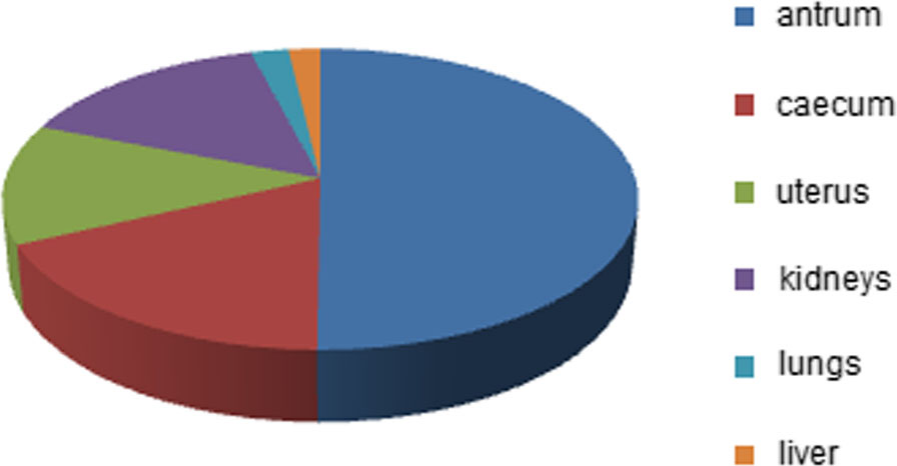
The relative amount of Ti in the investigated samples, %

The elemental analysis of the content of micro- and macro-elements in the tissues of rats was performed using the atomic emission spectrometer. All the tissue samples were obtained from the Wistar line rats, burdened with the suspension of titanium dioxide nanosize material in the abovementioned concentration for 30 days. It was determined (Fig. 9 and Table 1) that in these conditions, titanium dioxide gets accumulated in the tissues of animals with uneven distribution in different organs. Thus, the highest content of titanium (Ti) among the investigated samples was registered in the smooth muscle cells of the antrum preparations. There was rather high content of the mentioned element in the samples, obtained from circular smooth muscles of the caecum, uterus, and kidneys. The presence of Ti was registered in the lungs and liver, but in much lesser concentrations. The reason of considerable accumulation of Ti in smooth muscles of stomach and large intestines may be long-term presence of nanosize material of TiO_2_ along with the suspension and digestive masses in the cavities of the gastrointestinal tract. The increased content of this element in the kidney tissues allows assuming the possibility of releasing some amount of nanoparticles from the organism with urine.

**Table 1:** The concentration of Ti in the investigated samples

Type of sample	Content, mg/kg
Antrum	11.15
Caecum	3.97
Uterus	2.88
Kidneys	3.34
Lungs	0.49
Liver	0.41

## Conclusions

Thus, the results of the elemental analysis of the content of the micro- and macro-elements in the tissues of rats demonstrated that titanium dioxide in the form of nanosize material (10^3^–10^6^ atoms) with amphoteric properties is bioavailable for the cells of different organs with the highest accumulation of Ti in the smooth muscles of gastrointestinal tract. The tenzometric estimations of the spontaneous contractive activity of circular smooth muscles of the caecum and myometrium of rats, burdened with the suspension of TiO_2_, demonstrated that it was accompanied with the changes in amplitude–frequency and kinetic parameters of their spontaneous contractive activity, as indices of evaluating the status of the functioning of interstitial cells—the generators of pacemaking activity. The accumulation of titanium dioxide in smooth muscles of the caecum resulted in the considerable, compared to the control, increase in the frequency of spontaneous contractions and the significant decrease in the duration of the contraction–relaxation cycle as well as the decrease in the MU and AU indices of contractions (fourfold and sixfold, respectively) as the estimates of muscle functioning efficiency (contrary to MU index, AU index takes into consideration the contribution to the total decrease in the phases of contraction and relaxation as well as uneven nature of the occurrence of contraction units). The manifestation of some differences in the mechanisms of regulating the spontaneous contractions of the smooth muscles of the myometrium compared to the smooth muscles of the caecum was found in the decrease of the frequency of contractions and the duration of the contraction–relaxation cycle, as well as in the increase in the values of MU and AU indices in the group of rats, burdened with titanium dioxide for 30 days. The results of in vivo studies also demonstrated that nanosize titanium dioxide material modulates both the mechanisms of the input of extracellular Ca^2+^ ions and the mechanisms of decreasing the concentration of these cations in the smooth muscle cells of the caecum during the generation of the high potassium contraction and a considerable increase in the normalized maximal velocity of its components: the contraction phase and the relaxation phase. The sensitivity to the impact of TiO_2_ was also found in the cholinergic excitation of these muscles, namely, these may be the mechanisms, limiting the input of Ca^2+^ ions from the extracellular medium into the smooth muscle cells via potential-regulated calcium channels of the plasmatic membrane, which is indicated by the approximation of the ratio index of the values of phase and tonic components of acetyl choline-induced contraction to one point. The impact of titanium dioxide in the group of rats, burdened with this nanosize material, was accompanied with considerable impairment of the kinetics of forming the tonic component of the contraction of smooth muscles of myometrium, caused by oxytocin, the uterotonic peptide hormone.

## Abbreviations

5-HD: 5-Hydroxydecanoate
AC: Acetyl choline
AU: The index of contractions in Alexandria units
DMSO: Dimethylsulfoxide
MLC: Myosin light chains
MU: The index of contractions in Montevideo units
NKS: Normal Krebs solution
PTFE: Polytetrafluoroethylene
SMS: Smooth muscle stripes
TiO2: Titanium dioxide
